# Cytokinins Stimulate Plasmodesmatal Transport in Leaves

**DOI:** 10.3389/fpls.2021.674128

**Published:** 2021-05-31

**Authors:** Wilson Horner, Jacob O. Brunkard

**Affiliations:** ^1^Department of Plant and Microbial Biology, University of California, Berkeley, Berkeley, CA, United States; ^2^Plant Gene Expression Center, USDA Agricultural Research Service, Albany, CA, United States; ^3^Laboratory of Genetics, University of Wisconsin – Madison, Madison, WI, United States

**Keywords:** plasmodesmata, cytokinin, *AHP6*, *AHP5*, *AHK4*, *AHK3*, cell–cell signaling, phytohormones

## Abstract

Plant cells are connected by plasmodesmata (PD), nanoscopic channels in cell walls that allow diverse cytosolic molecules to move between neighboring cells. PD transport is tightly coordinated with physiology and development, although the range of signaling pathways that influence PD transport has not been comprehensively defined. Several plant hormones, including salicylic acid (SA) and auxin, are known to regulate PD transport, but the effects of other hormones have not been established. In this study, we provide evidence that cytokinins promote PD transport in leaves. Using a green fluorescent protein (GFP) movement assay in the epidermis of *Nicotiana benthamiana*, we have shown that PD transport significantly increases when leaves are supplied with exogenous cytokinins at physiologically relevant concentrations or when a positive regulator of cytokinin responses, *ARABIDOPSIS HISTIDINE PHOSPHOTRANSFER PROTEIN 5 (AHP5)*, is overexpressed. We then demonstrated that silencing cytokinin receptors, *ARABIDOPSIS HISTIDINE KINASE 3 (AHK3)* or *AHK4* or overexpressing a negative regulator of cytokinin signaling, *AAHP6*, significantly decreases PD transport. These results are supported by transcriptomic analysis of mutants with increased PD transport (*ise1–4*), which show signs of enhanced cytokinin signaling. We concluded that cytokinins contribute to dynamic changes in PD transport in plants, which will have implications in several aspects of plant biology, including meristem patterning and development, regulation of the sink-to-source transition, and phytohormone crosstalk.

## Introduction

Plasmodesmata (PD) are narrow, membrane-lined channels in plant cell walls that connect the cytosols of neighboring cells (Brunkard and Zambryski, 2017; Faulkner, 2018; Azim and Burch-Smith, 2020). Diverse cytosolic molecules move through PD, including metabolites, small RNAs, proteins up to ~80 kDa, and viruses. The size of molecules that can move through PD and the rate of trafficking through PD varies considerably during plant development and in response to physiological cues. However, little is known about how PD transport is regulated at the molecular level. To discover genetic pathways that coordinate PD transport, the Zambryski lab conducted forward genetic screens for mutants with increased or decreased PD transport at the mid-torpedo stage of *Arabidopsis embryogenesis* (Kim et al., 2002; Xu et al., 2012). These screens led to the discovery and characterization of five mutants so far: four with increased PD trafficking (*ise1–ise4*; Kobayashi et al., 2007; Stonebloom et al., 2009; Burch-Smith and Zambryski, 2010; Burch-Smith et al., 2011; Brunkard et al., 2020) and one with decreased PD trafficking (*dse1*; Xu et al., 2012).

To identify pathways that could contribute to the increased PD transport phenotype observed in *ise* mutants, we took a comparative transcriptomic approach. Previously, we used this approach to discover that chloroplast retrograde signaling (Burch-Smith et al., 2011; Burch-Smith and Zambryski, 2012; Brunkard et al., 2013) and target of rapamycin (TOR) signaling (Brunkard et al., 2020) coordinate PD transport in embryos and leaves. One of the most strongly repressed genes in both *ise1* and *ise2* embryos is *ARABIDOPSIS HISTIDINE PHOSPHOTRANSFER PROTEIN 6* (*AHP6*; Burch-Smith et al., 2011). *AHP6* is expressed during wild-type embryogenesis from the heart stage through the torpedo stage; later in development, *AHP6* is most strongly expressed in inflorescence and root meristems (Bishopp et al., 2011; Besnard et al., 2014). In both *ise1* and *ise2*, *AHP6* expression is reduced by >20-fold at the mid-torpedo stage of development compared to wild-type plants, one of the most strongly repressed genes in these transcriptomes (Burch-Smith et al., 2011).

*ARABIDOPSIS HISTIDINE PHOSPHOTRANSFER PROTEIN 6* is a member of the *AHP* family (Hutchison et al., 2006; Mähönen et al., 2006), which is composed of histidine phosphotransfer proteins that mediate responses to a phytohormone, cytokinin, *via* a two-component system (Hwang and Sheen, 2001). Briefly, cytokinins directly bind to and stimulate a family of histidine kinases (*ARABIDOPSIS HISTIDINE KINASE (AHK) 2*, *AHK3*, and *AHK4*) that phosphorylate AHPs, which then transfer phosphorylation and thus activate *ARABIDOPSIS RESPONSE REGULATORs (ARRs)*, a large family of diverse transcription factors (Müller and Sheen, 2007). Cytokinins regulate diverse developmental and physiological processes, with especially important roles in cell fate and proliferation (Amasino, 2005; Hwang et al., 2012; Wybouw and De Rybel, 2019). *AHP6* is unique in this pathway because it is a pseudo-histidine phosphotransfer protein with a mutation in the conserved histidine residue that prevents it from relaying the phosphorylation to response regulators (Mähönen et al., 2006). Instead of facilitating cytokinin signaling, *AHP6* interferes with the phosphorelay and attenuates cytokinin responses. Detailed studies of *AHP6* have revealed that it is transcriptionally induced by another phytohormone, auxin, which often antagonizes cytokinin signaling (Bishopp et al., 2011). After transcription and translation, the small (17.9 kDa) cytosolic *AHP6* protein freely moves to neighbor cells *via* PD, effectively establishing an inhibitory field that limits cytokinin responses and thereby locally enhances the formation of auxin maxima (Bishopp et al., 2011; Besnard et al., 2014). In meristems, the mobile *AHP6* signal helps to define boundaries and establish robust developmental patterning (Besnard et al., 2014). In embryos, *AHP6* is expressed primarily in cotyledons and differentiating vasculature (Bishopp et al., 2011), but given the small size of *AHP6*, we suspect that the *AHP6* protein may spread to an even larger domain when it is briefly transcriptionally induced during the heart-to-torpedo stages.

Phytohormones can play crucial roles in regulating PD transport during plant development and physiological responses to biotic and abiotic stresses (Lee, 2014; Brunkard et al., 2015b). For example, salicylic acid (SA) triggers membrane remodeling and callose deposition in the cell wall surrounding PD, limiting PD trafficking in response to pathogen infection (Lee et al., 2011; Wang et al., 2013; Lim et al., 2016; Huang et al., 2019). Auxin also stimulates callose deposition in the cell wall surrounding PD, restricting PD transport during developmental transitions, such as lateral root formation (Benitez-Alfonso et al., 2009; Han et al., 2014). Little is known about the connections between other phytohormones and PD, although there is evidence that other hormones can at least conditionally regulate PD trafficking. For example, abscisic acid promotes dormancy in *Populus* buds during winter, in part by decreasing PD transport to isolate buds from growth signals (Tylewicz et al., 2018); gibberellins antagonize abscisic acid signaling and can therefore impact PD transport, at least in this context (Singh et al., 2019). Cytokinins can stimulate PD formation in some circumstances (Ormenese et al., 2006), but it is not known whether cytokinins dynamically impact PD transport in plant cells. In this study, using an established model system for functional studies of PD transport, the leaf epidermis of *Nicotiana benthamiana*, we directly tested how the cytokinin signaling network affects PD transport.

## Materials and Methods

### Plant Growth Conditions

For *trans*-Zeatin application experiments, *N. benthamiana* (accession Nb-1) plants were grown in a greenhouse at 22°C and in 16-h daylengths for 4 weeks prior to infiltration. For transient overexpression experiments, growth conditions were identical, but plants were grown for 4 or 5 weeks before infiltration, depending on the size of the plant. For virus-induced gene silencing (VIGS) experiments, plants were grown in autoclaved soil and in isolation from other plants to prevent the presence of pathogens and pests at 22°C and in 16-h daylengths for 3 weeks prior to silencing. All plants used in the VIGS experiments were photographed prior to infiltration to assess phenotypic differences among *AHK3*-, *AHK4*-, and mock β-glucuronidase (*GUS*)-silenced plants and validate effective silencing with *PHYTOENE DESATURASE* (*PDS*)-silenced plants. As previously described (Brunkard et al., 2015a), plants that have effectively silenced *PDS* exhibit photobleached leaves.

### Cloning Silencing Triggers

Silencing triggers were cloned as previously described (Brunkard et al., 2015a). Briefly, RNA was isolated from *N. benthamiana*, Nb-1, with the Spectrum Plant Total RNA kit (Sigma-Aldrich, St. Louis, MO, United States), treating RNA with on-column DNase I digestion (New England Biolabs, Ipswich, MA, United States). Complementary DNA (cDNA) was synthesized from isolated RNA using random hexamers and SuperScript III reverse transcriptase (Fisher Scientific, Waltham, MA, United States). Silencing triggers were amplified with Phusion DNA polymerase (New England Biolabs). Triggers and the TRV2 plasmid pYL156 were digested with XbaI and XhoI (New England Biolabs) and ligated with Promega T4 DNA ligase (Fisher Scientific). Ligations were transformed into XL1-Blue *Escherichia coli*, were mini-prepped (New England Biolabs, Ipswich, MA, United States), and were Sanger sequenced to confirm insertion sequences. The *AHK4* trigger was cloned with oligonucleotides 5'-gat TCT AGA AAC TAT GGA GGA ACG GG-3' and 5'-gat ctc GAG GTT TCA TTA TCA CCG C-3' to silence the two *AHK4* homologues, *Niben101Scf08855g02013* and *Niben101g09260g03002*. The *AHK3* trigger was cloned with oligonucleotides 5'-cat TCT AGA TGT GAC ACA ACA AGA TTA TGT C-3' and 5'-gat ctC GAG CAA TAG AAG GAC CAA C-3' to silence the two *AHK3* homologues, *Niben10103911g05013* and *Niben101Scf02711g00003*.

### Cloning AHP Overexpression Constructs

As with the silencing triggers, *AHP5* and *AHP6* were amplified with Phusion DNA polymerase (New England Biolabs) from *N. benthamiana* cDNA that was synthesized as above. *AHP5* was cloned using oligonucleotides 5'-aattacaggcctcccgggaccATGAACACCATCGTCGTT-3' and 5'-TTCGCTTCCTGAccc CTAATTTATATCCACTTGAGGAATT-3', while *AHP6* was cloned using oligonucleotides 5'-aattacaggcctcccgggaccATGTTGGGGTTGGGTGTG-3' and 5'-TTCGCTTCCTGAcccCTACATTGGATATCTGACTCCTGC-3'. All oligonucleotides contained 15 bp of homology compatible with the SmaI digestion site of binary vectors containing a CaMV 35S promoter, TMV Omega enhancer sequence, and CaMV 35S terminator for transient gene expression. The plasmid was digested with SmaI, and both genes were treated with T5 DNA exonuclease (New England Biolabs) as previously described (Xia et al., 2019). These reactions were used to transform chemically competent DH10B *E. coli*, were mini-prepped, and were Sanger sequenced to confirm insertion sequences.

### Agroinfiltration

*Agrobacterium tumefaciens* strain, GV3101, was grown overnight in a lysogeny broth medium at 28°C, 250 rpm, with kanamycin, gentamicin, and rifampicin (each at 50 mg ml^−1^). Cultures were centrifuged at ×700 *g* for 10 min and then resuspended in an infiltration medium [10 mM MgCl_2_, 10 mM 2-(N-morpholino)ethanesulfonic acid (MES), and 200 μM acetosyringone, pH 5.6, adjusted with KOH] to OD_600nm_ = 1.0. Agrobacteria were then left to induce virulence at room temperature for 2–4 h with gentle shaking prior to infiltration. Immediately before infiltrating, cultures were then further diluted in infiltration media to OD_600nm_ = 10^−5^ for green fluorescent protein (GFP) movement assays or OD_600nm_ = 0.1 for overexpression. Cultures were left at OD_600nm_ = 1.0 for VIGS, as previously described (Brunkard et al., 2015a).

Briefly, for VIGS experiments, the first two true leaves of *N. benthamiana* plants were infiltrated with equal induced inocula of *A. tumefaciens* carrying the previously described binary vectors: pYL192 (which expresses the TRV1 subgenome) and pYL156 (which expresses the TRV2 subgenome with silencing triggers). A TRV2-*GUS* trigger was used as a negative control for VIGS, and a TRV2-*NbPDS* trigger was used as a positive control for VIGS. About 14 days after infiltration of the VIGS inocula, the fourth leaf of each plant was infiltrated with an induced inoculum containing the *35S_PRO_:GFP* binary vector diluted to OD_600nm_ = 10^−5^. Each plant was left under normal growing conditions (22°C and 16-h daylengths) for 48 h prior to GFP movement assays (described below).

For overexpression experiments, the fourth leaf from 4-to-5-week-old *N. benthamiana* plants was infiltrated with an induced inoculum containing the *35S_PRO_:GFP* binary vector diluted to OD_600nm_ = 10^−5^ and the transient overexpression vector diluted to OD_600nm_ = 0.1, with an empty transient overexpression plasmid used as a negative control. These plants were left under normal growing conditions (22°C and 16-h daylengths) for 72 h prior to GFP movement assays (described below).

### *Trans*-Zeatin Infiltration

A 1 mM stock solution of *trans*-Zeatin (Cayman Chemical Company) in DMSO was diluted to several concentrations (1.0 nM, 10 nM, and 100 nM) in infiltration media, with the infiltration media containing no *trans*-Zeatin used as a negative control. The *35S_PRO_:GFP* binary vector was then diluted to OD_600nm_ = 10^−5^ in these solutions and infiltrated into the fourth leaf from 4-week-old *N. benthamiana* plants, as above. These plants were left under normal growing conditions (22°C and 16-h daylengths) for 48 h prior to GFP movement assays (described below).

### Assaying PD Transport With GFP Transformation

**Figure 1:**
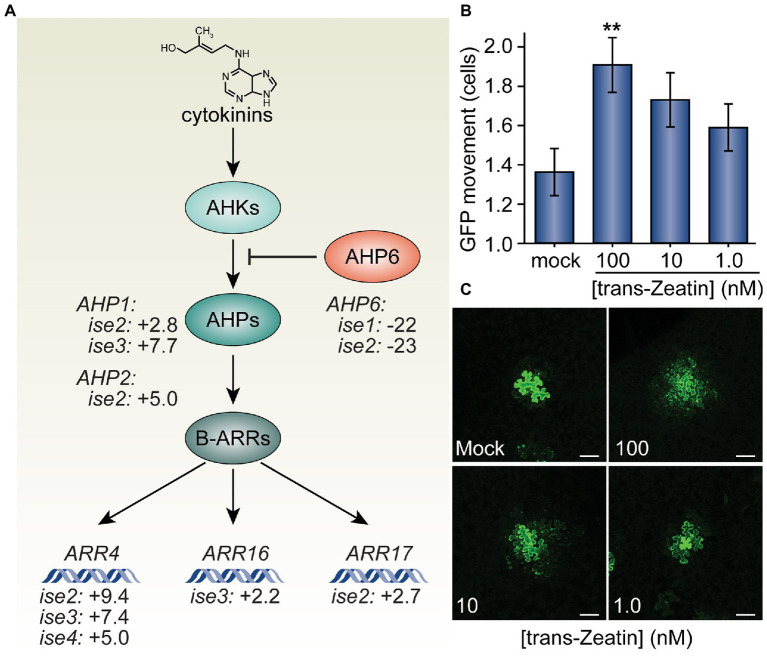
*Trans*-Zeatin (cytokinin) stimulates plasmodesmata (PD)-mediated green fluorescent protein (GFP) movement in *Nicotiana benthamiana* leaves. **(A)** Cytokinins (e.g., *trans*-Zeatin, whose structure is shown) stimulate the cytokinin receptors, *ARABIDOPSIS HISTIDINE KINASES* (*AHK*s, light teal), to phosphorylate *ARABIDOPSIS HISTIDINE PHOSPHOTRANSFER PROTEINS* (*AHP*s, teal), which transfer phosphorylation to B-class *ARABIDOPSIS RESPONSE REGULATORS* (*ARR*s, dark teal), which then promote transcription of cytokinin response genes, including the A-type *ARR* genes, *ARR4*, *ARR16*, and *ARR17*. *AHP6* (orange) acts as a decoy *AHP*, preventing phosphorylation of *AHP*s and thus antagonizing cytokinin signaling. Transcriptomic analysis of four mutants with increased PD trafficking, *ise1* through *ise4*, revealed the induction of *AHP* genes that promote cytokinin signaling, induction of A-type *ARR* genes that indicate elevated cytokinin responses, and repression of the *AHP6* gene that inhibits cytokinin signal transduction (Burch-Smith et al., 2011; Brunkard et al., 2020). Fold-changes in mRNA levels of these genes are indicated. **(B)** Infiltration of 100 nM solutions of *trans*-Zeatin significantly increased PD transport (*n* = 53, ^**^p < 0.01; error bars indicate SEM). 10 or 1.0 nM *trans*-Zeatin somewhat increased PD transport, but not to statistically significant thresholds (*n* = 51 or *n* = 56, respectively). **(C)** Representative confocal images of transformed *N. benthamiana* cells for different treatments of the *trans*-Zeatin show the range of GFP movement from transformed cells. In mock-treated leaves, GFP rarely moved 1–2 cells beyond the transformed cell. After applying *trans*-Zeatin, GFP movement tended to increase, often moving to three cells or more beyond the transformed cell in leaves treated with 100 nM *trans*-Zeatin. The fourth leaf from 4-week-old *N. benthamiana* plants was used for each experiment; white scale bars = 100 μm.

Plasmodesmata movement assays were performed using the fourth leaf from either 4-week-old (for *trans*-Zeatin treatment experiments) or 4-to-5-week-old (for VIGS and overexpression experiments) *N. benthamiana* plants. GFP movement assays were conducted as previously described (Brunkard et al., 2015a; Figure 1), observing GFP movement in only the proximal 25% of the leaf. Briefly, leaves were infiltrated with very low inocula of *Agrobacterium* (OD_600nm_ < 10^−4^) carrying a binary vector to transform cells to express GFP under the CaMV 35S promoter. Only a handful of individual, isolated cells are transformed by the low inocula of *Agrobacterium*. About 48 h after agroinfiltration, the genetically transformed cells show bright GFP fluorescence and are surrounded by “rings” of cells with lower fluorescence, indicating that GFP has moved into these cells *via* PD. In this study, we report the greatest distance, in numbers of cells, that GFP has spread.

In all experiments, leaves that were previously infiltrated with the *35S_PRO_:GFP* vector were infiltrated with water immediately prior to harvesting. For VIGS experiments, leaves were harvested from plants infiltrated with media containing the *35S_PRO_:GFP* vector 48 h after infiltration. For *trans*-Zeatin experiments, leaves were similarly harvested from plants infiltrated with media containing *trans*-Zeatin and the *35S_PRO_:GFP* vector 48 h after infiltration. For overexpression experiments, leaves were harvested from plants infiltrated with media containing the *35S_PRO_:GFP* vector 72 h after infiltration. For all conditions, small sections were cut from each infiltrated leaf (~1 × 2 cm), mounted abaxial side up on microscope slides, and imaged (as shown in “Microscopy” below).

For each experiment, at least 5, and as many as 24, plants were assayed for each condition per replicate, and each experiment was replicated three times, resulting in each experiment being conducted in at least 70 plants. GFP movement from 1 to 5 randomly selected transformed cells per plant was observed, depending on how many transformed cells were found in the section used for microscopy. The movement was scored by counting the distance in rings of cells to which GFP had moved from the originally transformed cell (e.g., no movement was scored as zero, since GFP remained only in the transformed cell; movement into one or all cells immediately touching the originally transformed cell but none beyond was scored as one; and so on). The total number of cells containing GFP was also counted for each sample.

### Microscopy

GFP was observed in the epidermis of *N. benthamiana* leaves using a Leica DM6 CS confocal laser scanning microscope, with settings as described by Brunkard et al. (2015a). To ensure no artifacts were introduced during microscopy, identical settings (such as laser strength, gain, emission filters, and aperture) were used in all experiments, and samples were imaged in randomized order to avoid any bias during experimentation. All movement assays were scored by both authors to ensure reproducibility.

### Quantification and Statistical Analysis

Movement assay results are presented as the average movement of GFP and SEM. Differences in GFP movement between two given conditions were compared using unpaired heteroscedastic Student’s *t*-tests in Excel, with *p* < 0.05 being considered significantly different.

## Results

Based on the previous finding that the cytokinin signaling inhibitor, *AHP6*, is severely transcriptionally repressed in *ise1* and *ise2* (Burch-Smith et al., 2011), we explored whether the transcriptomes of *ise* mutants reveal any clear changes in the gene expression that could reflect enhanced cytokinin signaling, which would support the hypothesis that cytokinin signaling could contribute to the *ise* phenotype. Indeed, *ise2* shows several additional signatures of elevated cytokinin signaling, including a 9-fold increase in the messenger RNA (mRNA) level of a standard transcriptional reporter for cytokinin responses, *ARR4* (*ARABIDOPSIS RESPONSE REGULATOR 4*), and >3-fold induction of cytokinin phosphorelay proteins, *AHP1* and *AHP2*, that promote cytokinin responses. The cytokinin response reporter gene, *ARR4*, is also induced >5-fold in both *ise3* and *ise4*, suggesting that elevated cytokinin signaling could be a common feature of *ise* mutants.

### Cytokinin Can Stimulate PD Movement in Leaves

Given the evidence of enhanced cytokinin signaling and increased PD transport in the *ise* mutants, we next tested whether exogenous application of cytokinin is sufficient to increase PD transport. To this end, we infiltrated leaves of 4-week-old *N. benthamiana* plants with infiltration media containing a low inoculum of *Agrobacterium* (OD_600nm_ < 10^−4^) carrying a *35S_PRO_:GFP* binary vector and a range of concentrations (1.0, 10, or 100 nM) of the cytokinin *trans*-Zeatin (Letham and Miller, 1965). Infiltration media with no *trans*-Zeatin was used as a negative control. About 48 h post-infiltration, we excised sections of infiltrated leaves, imaged GFP foci with confocal microscopy, and statistically analyzed the results to quantitatively assess GFP movement. The final results are expressed as the maximal distance that GFP had spread from the transformed cell into neighboring cells.

We found that higher concentrations of *trans*-Zeatin correspondingly increased the movement of GFP in leaves (Figure 1). On average, GFP moved 1.36 ± 0.12 cells from the transformed cell in the presence of no exogenous *trans*-Zeatin. With the addition of 100 nM of *trans*-Zeatin, GFP moved 1.91 ± 0.14 cells from the transformed cell; when compared with results from the control, we found that this difference is statistically significant (*n* = 53, *p* < 0.01). Even with smaller concentrations of *trans*-Zeatin application, GFP movement also apparently increased, though to proportionately smaller degrees (1.73 ± 0.14 cells with 10 nM of *trans*-Zeatin, 1.59 ± 0.12 cells with 1.0 nM of *trans*-Zeatin). Overall, these results demonstrate that cytokinins promote PD movement.

### Silencing Expression of Cytokinin Receptor Genes Reduces PD Transport

Following these results, we took a genetic approach and tested whether silencing the expression of genes directly involved in cytokinin sensing would result in lowered PD movement. Using VIGS, we silenced *AHK3* and *AHK4*, which encode cytokinin receptors that initiate the cytokinin-*AHK*-*AHP*-*ARR* signal transduction pathway in plant cells. We used a TRV2-*GUS* trigger as a negative control for silencing and a TRV2-*NbPDS* trigger as a positive control for silencing. After plants were infiltrated with the VIGS constructs, we allowed 2 weeks for silencing to establish before infiltrating leaves on each plant with the same *35S_PRO_:GFP* construct used in other experiments. Immediately prior to this, we took photographs of each plant to document phenotypic differences among conditions. We observed no obvious morphological or physiological differences between mock silenced and *AHK*-silenced plants, so we experimented in the same manner as above for the *trans*-Zeatin experiments.

**Figure 2:**
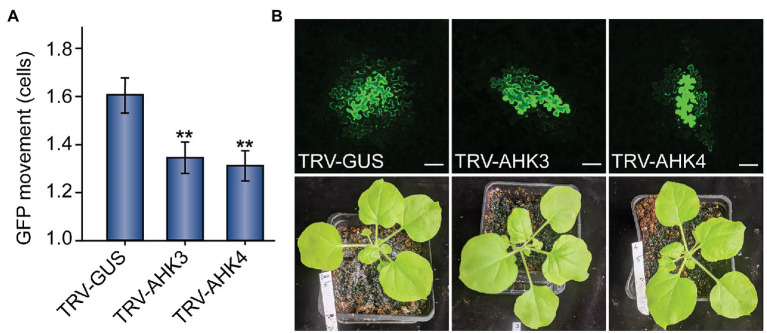
Virus-induced gene silencing (VIGS) of cytokinin receptor expression reduces PD-mediated GFP movement in *N. benthamiana* leaves. **(A)** Compared to control TRV-GUS plants, GFP movement from transformed cells was significantly lowered in plants where the cytokinin receptors *AHK3* or *AHK4* were silenced (*n* = 124, ^**^p < 0.01; or *n* = 133, ^**^p < 0.01; respectively; error bars indicate SEM). **(B)** Representative confocal images of transformed *N. benthamiana* cells and examples of 5-week-old VIGS-treated plants indicate that GFP movement was reduced in *AHK3* and *AHK4*-silenced plants, but there were no obvious phenotypic effects on the plants themselves. In mock-treated TRV-GUS plants, GFP typically moved 1–3 cells beyond the transformed cell; in *AHK3* and *AHK4*-silenced plants, GFP movement rarely exceeded two cells. The fourth leaf from 5-week-old *N. benthamiana* plants was used for each experiment; white scale bars = 100 μm.

In both *AHK3*‐ and *AHK4*-silenced plants, we observed a significant decrease in GFP movement relative to the *GUS* mock treatment (Figure 2). Whereas GFP moved 1.61 ± 0.07 cells from the transformed cell in mock-silenced (TRV-GUS) plants, GFP movement in *AHK3*-silenced plants was reduced to 1.31 ± 0.07 cells and to 1.35 ± 0.06 cells in *AHK4*-silenced plants. We found that both results were statistically significant (*n* = 124, *p* < 0.01; *n* = 133, *p* < 0.01). These findings further bolstered the hypothesis that the regulation of PD transport is intimately linked to the cytokinin signaling pathway.

### *AHP5* and *AHP6* Antagonistically Regulate PD Transport in Leaves

Given the findings regarding the influence of upstream members of the cytokinin signaling pathway on GFP movement, we asked whether overexpressing proteins downstream of *AHK*s in the cytokinin signaling pathway would alter PD transport. To test this hypothesis, we cloned *AHP5* and *AHP6* into binary vectors for transient expression in *N. benthamiana* leaves. Agrobacteria carrying *35S_PRO_:AHP5* or *35S_PRO_:AHP6* were then co-infiltrated with *35S_PRO_:GFP* into *N. benthamiana* leaves.

**Figure 3:**
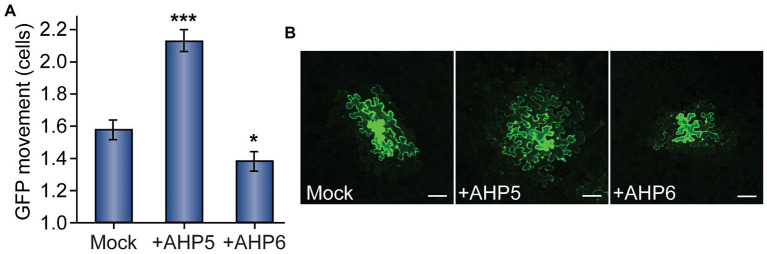
Cytokinin phosphorelay proteins *AHP5* and *AHP6* regulate PD transport. **(A)** Compared to mock-infiltrated plants, GFP movement from transformed cells significantly increased in leaves overexpressing *AHP5* (*n* = 141, ^***^p < 0.001; error bars indicate SEM). Overexpressing the negative regulator of cytokinin signaling, *AHP6*, had the opposite effect, decreasing PD transport in leaves (*n* = 161, ^*^p < 0.05; error bars indicate SEM). **(B)** Representative confocal images of transformed *N. benthamiana* cells demonstrate the difference in GFP movement between conditions. GFP moved 1–3 cells beyond the transformed cell in mock-treated leaves. In leaves overexpressing *AHP5*, GFP regularly moved more than two cells and often as many as four cells from the transformed cell. In leaves overexpressing *AHP6*, GFP movement was confined to one, or sometimes two, cell(s) beyond the transformed cell. The fourth leaf from 4-to-5-week-old *N. benthamiana* plants was used for each experiment; white scale bars = 100 μm.

When compared to control plants infiltrated with empty vector, we found that leaves overexpressing *AHP5* exhibited significantly increased levels of PD transport, whereas leaves overexpressing *AHP6* exhibited significantly decreased levels of PD transport (Figure 3). In leaves agroinfiltrated with empty vector, GFP moved 1.58 ± 0.07 cells from the transformed cell vs. 2.13 ± 0.07 for plants overexpressing *AHP5* and 1.38 ± 0.06 for plants overexpressing *AHP6*. The change in GFP movement was significant for both *AHP5*-overexpressing plants (*n* = 141, *p* < 0.001) and *AHP6*-overexpressing plants (*n* = 161, *p* < 0.05) relative to control plants. Together, these results indicate that manipulating the expression of different members of the cytokinin signaling pathway phosphorelay chain directly impacts the rate of PD transport.

## Discussion

In this study, we provide evidence that cytokinins can stimulate intercellular trafficking through PD in plants. We demonstrated that manipulating the cytokinin signaling pathway produces consistent observable effects on GFP movement in the leaf epidermal tissue. Directly infiltrating cytokinins increased the rate of GFP movement, as did overexpression of the gene encoding a phosphotransfer protein, *AHP5*, that promotes cytokinin responses. Conversely, overexpressing *AHP6*, which encodes a protein that lacks the phosphotransfer capability of *AHP5* and thus suppresses cytokinin responses, or silencing *AHK3* or *AHK4*, which encodes two cytokinin receptors, decreased PD-mediated GFP movement. Given the transcriptomic signs that cytokinin signaling is enhanced in *ise* mutants, it is plausible that increased cytokinin levels contribute to the elevated PD transport observed in *ise* embryos.

Previous studies in non-model systems demonstrated that cytokinin treatment can induce the *de novo* formation of PD in cell walls (Ormenese et al., 2006), but this is the only direct experimental evidence that cytokinin signaling affects PD trafficking. Cytokinins can now be added to the growing list of physiological and developmental cues that dynamically regulate PD transport, including auxin, SA, abscisic acid, light, and the circadian clock, metabolic status, and oxidative stress, among others. Admittedly, in this report, we have not determined how cytokinins promote trafficking through PD. The best-defined mechanisms that mediate changes in PD trafficking are the reversible hyperaccumulation of callose in the cell wall surrounding PD, which is believed to occlude the PD channel and prevent trafficking, or *de novo* PD biogenesis, which is consistently correlated with increased PD transport. We hypothesize that cytokinins could act through multiple mechanisms to alter PD transport. For example, cytokinin could stimulate rapid *de novo* PD biogenesis, which will require further investigation in the future. We should note that there are more PD in the cell walls of *ise1* and *ise2* embryos; if cytokinin indeed does stimulate the formation of new PD in cell walls, the additional PD in *ise1*/*ise2* embryos could correlate with the induction of transcriptional responses to cytokinin in these mutants.

Although a relationship between cytokinin levels and PD transport in leaves has not been previously explored, cytokinin is known to impact sink-source relations in plants (Peleg et al., 2011; Kieber and Schaller, 2014). As young leaves develop, they transition from rapidly-growing “sinks” that import sugars to mature “sources” that export sugars for long-distance transport *via* the phloem (Turgeon, 2010). PD transport rapidly decreases during the sink-to-source transition, which is thought to contribute to the sink-to-source transition by limiting the diffusive backflow of sugars from the phloem into the exporting source leaf (Roberts et al., 1997, 2001; Imlau et al., 1999; Brunkard, 2020; Brunkard et al., 2020). Genetic and physiological experiments have shown that cytokinins increase so-called “sink strength” in leaves, the rate of sugar import into growing leaves. For example, tobacco transformed to overexpress cytokinin oxidases, which degrade cytokinins and thus reduce cytokinin signaling, decreased the concentrations of glucose, fructose, and sucrose by as much as 10-fold in sink leaves without comparably affecting the sugar concentrations in source leaves (Werner et al., 2008). While the defect in sink strength is likely due to multiple pathways impacted by cytokinin signaling, we speculate that PD transport could be limited in cytokinin-deficient plants, effectively reducing the rate of phloem import to sink leaves.

Plasmodesmata transport dynamics are especially crucial for patterning in the shoot apical meristem (SAM; Rinne and Van der Schoot, 1998; Gisel et al., 1999; Kitagawa and Jackson, 2019). Multiple transcription factors that determine whether SAM cells proliferate, differentiate, or remain quiescent readily move between cells *via* PD, including the homeobox proteins Knotted1 (Kn1, sometimes called SHOOT MERISTEMLESS or STM in *Arabidopsis*; Lucas et al., 1995) and WUSCHEL (WUS; Yadav et al., 2011). The hormones that specify cell fate in the meristem, especially cytokinin and auxin, which act antagonistically to regulate meristem size and organ initiation, can also move through PD (Kitagawa and Jackson, 2019). Kn1 and WUS maintain stem cell fates in the meristem partly by stimulating cytokinin biosynthesis; the cytokinins then move through PD to neighboring cells, presumably forming a concentration gradient. In contrast, as auxin maxima form, auxin response factors drive the expression of *AHP6* to locally prevent cytokinin signal transduction (Besnard et al., 2014). Like the transcription factors and hormones, *AHP6* also moves through PD, creating a zone of cells that are “immune” to the WUS-Kn1-promoted cytokinin biosynthesis (Besnard et al., 2014). The discovery that supplying cells with cytokinin is sufficient to increase PD transport suggests that cytokinin-PD signaling should be considered in models of cell–cell communication at the SAM. Given the intricate balance of molecules, ranging from metabolites and hormones to transcription factors and small RNAs, that move through PD in the SAM, we expect that reevaluation of SAM dynamics in light of cytokinin-PD signaling could open exciting new avenues for future research.

## Data Availability Statement

The original contributions presented in the study are included in the article/supplementary material, further inquiries can be directed to the corresponding author.

## Author Contributions

WH and JB designed the project, analyzed the data, and wrote the manuscript. WH performed the experiments. JB initiated and supervised the project. All authors contributed to the article and approved the submitted version.

### Conflict of Interest

The authors declare that the research was conducted in the absence of any commercial or financial relationships that could be construed as a potential conflict of interest.

## References

[ref1] Amasino R. (2005). 1955: Kinetin arrives. The 50th anniversary of a new plant hormone. Plant Physiol. 138, 1177–1184. 10.1104/pp.104.900160, PMID: 16009993 PMC1176392

[ref2] Azim M. F. Burch-Smith T. M. (2020). Organelles-nucleus-plasmodesmata signaling (ONPS): an update on its roles in plant physiology, metabolism and stress responses. Curr. Opin. Plant Biol. 58, 48–59. 10.1016/j.pbi.2020.09.005, PMID: 33197746

[ref3] Benitez-Alfonso Y. Cilia M. San Roman A. Thomas C. Maule A. Hearn S.. (2009). Control of *Arabidopsis* meristem development by thioredoxin-dependent regulation of intercellular transport. Proc. Natl. Acad. Sci. U. S. A. 106, 3615–3620. 10.1073/pnas.0808717106, PMID: 19218459 PMC2651306

[ref4] Besnard F. Refahi Y. Morin V. Marteaux B. Brunoud G. Chambrier P.. (2014). Cytokinin signalling inhibitory fields provide robustness to phyllotaxis. Nature 505, 417–421. 10.1038/nature12791, PMID: 24336201

[ref5] Bishopp A. Help H. El-Showk S. Weijers D. Scheres B. Friml J.. (2011). A mutually inhibitory interaction between auxin and cytokinin specifies vascular pattern in roots. Curr. Biol. 21, 917–926. 10.1016/j.cub.2011.04.017, PMID: 21620702

[ref6] Brunkard J. O. (2020). Exaptive evolution of target of rapamycin signaling in multicellular eukaryotes. Dev. Cell 54, 142–155. 10.1016/j.devcel.2020.06.022, PMID: 32649861 PMC7346820

[ref7] Brunkard J. O. Burch-Smith T. M. Runkel A. M. Zambryski P. C. (2015a). Investigating plasmodesmata genetics with virus-induced gene silencing and an agrobacterium-mediated GFP movement assay. Methods Mol. Biol. 1217, 185–198. 10.1007/978-1-4939-1523-1_13, PMID: 25287205

[ref8] Brunkard J. O. Runkel A. M. Zambryski P. C. (2013). Plasmodesmata dynamics are coordinated by intracellular signaling pathways. Curr. Opin. Plant Biol. 16, 614–620. 10.1016/j.pbi.2013.07.007, PMID: 23978390 PMC3828052

[ref9] Brunkard J. O. Runkel A. M. Zambryski P. C. (2015b). The cytosol must flow: intercellular transport through plasmodesmata. Curr. Opin. Cell Biol. 35, 13–20. 10.1016/j.ceb.2015.03.003, PMID: 25847870

[ref10] Brunkard J. O. Xu M. Regina Scarpin M. Chatterjee S. Shemyakina E. A. Goodman H. M.. (2020). TOR dynamically regulates plant cell-cell transport. Proc. Natl. Acad. Sci. U. S. A. 117, 5049–5058. 10.1073/pnas.1919196117, PMID: 32051250 PMC7060719

[ref11] Brunkard J. O. Zambryski P. C. (2017). Plasmodesmata enable multicellularity: new insights into their evolution, biogenesis, and functions in development and immunity. Curr. Opin. Plant Biol. 35, 76–83. 10.1016/j.pbi.2016.11.007, PMID: 27889635

[ref12] Burch-Smith T. M. Brunkard J. O. Choi Y. G. Zambryski P. C. (2011). Organelle-nucleus cross-talk regulates plant intercellular communication via plasmodesmata. Proc. Natl. Acad. Sci. U. S. A. 108, E1451–E1460. 10.1073/pnas.1117226108, PMID: 22106293 PMC3251100

[ref13] Burch-Smith T. M. Zambryski P. C. (2010). Loss of increased size exclusion limit (ise)1 or ise2 increases the formation of secondary plasmodesmata. Curr. Biol. 20, 989–993. 10.1016/j.cub.2010.03.064, PMID: 20434343 PMC2902234

[ref14] Burch-Smith T. M. Zambryski P. C. (2012). Plasmodesmata paradigm shift: regulation from without versus within. Annu. Rev. Plant Biol. 63, 239–260. 10.1146/annurev-arplant-042811-105453, PMID: 22136566

[ref15] Faulkner C. (2018). Plasmodesmata and the symplast. Curr. Biol. 28, R1374–R1378. 10.1016/j.cub.2018.11.004, PMID: 30562524

[ref16] Gisel A. Barella S. Hempel F. D. Zambryski P. C. (1999). Temporal and spatial regulation of symplastic trafficking during development in *Arabidopsis thaliana* apices. Development 126, 1879–1889. 10.1242/dev.126.9.1879, PMID: 10101122

[ref17] Han X. Hyun T. K. Zhang M. Kumar R. Koh E. ji Kang B. H. (2014). Auxin-callose-mediated plasmodesmal gating is essential for tropic auxin gradient formation and signaling. Dev. Cell 28, 132–146. 10.1016/j.devcel.2013.12.008, PMID: 24480642

[ref18] Huang D. Sun Y. Ma Z. Ke M. Cui Y. Chen Z.. (2019). Salicylic acid-mediated plasmodesmal closure via remorin-dependent lipid organization. Proc. Natl. Acad. Sci. U. S. A. 116, 21274–21284. 10.1073/pnas.1911892116, PMID: 31575745 PMC6800329

[ref19] Hutchison C. E. Li J. Argueso C. Gonzalez M. Lee E. Lewis M. W.. (2006). The *Arabidopsis* histidine phosphotransfer proteins are redundant positive regulators of cytokinin signaling. Plant Cell 18, 3073–3087. 10.1105/tpc.106.045674, PMID: 17122069 PMC1693944

[ref20] Hwang I. Sheen J. (2001). Two-component circuitry in *Arabidopsis* cytokinin signal transduction. Nature 413, 383–389. 10.1038/35096500, PMID: 11574878

[ref21] Hwang I. Sheen J. Müller B. (2012). Cytokinin signaling networks. Annu. Rev. Plant Biol. 63, 353–380. 10.1146/annurev-arplant-042811-105503, PMID: 22554243

[ref22] Imlau A. Truernit E. Sauer N. (1999). Cell-to-cell and long-distance trafficking of the green fluorescent protein in the phloem and symplastic unloading of the protein into sink tissues. Plant Cell 11, 309–322. 10.1105/tpc.11.3.309, PMID: 10072393 PMC144181

[ref23] Kieber J. J. Schaller G. E. (2014). Cytokinins. Arabidopsis Book 12:e0168. 10.1199/tab.0168, PMID: 24465173 PMC3894907

[ref24] Kim I. Hempel F. D. Sha K. Pfluger J. Zambryski P. C. (2002). Identification of a developmental transition in plasmodesmatal function during embryogenesis in *Arabidopsis thaliana*. Development 129, 1261–1272. 10.1242/dev.129.5.1261, PMID: 11874921

[ref25] Kitagawa M. Jackson D. (2019). Control of meristem size. Annu. Rev. Plant Biol. 70, 269–291. 10.1146/annurev-arplant-042817-040549, PMID: 31035828

[ref26] Kobayashi K. Otegui M. S. Krishnakumar S. Mindrinos M. Zambryski P. (2007). Increased size exclusion limit2 encodes a putative DEVH box RNA helicase involved in plasmodesmata function during Arabidopsis embryogenesis. Plant Cell 19, 1885–1897. 10.1105/tpc.106.045666, PMID: 17601829 PMC1955720

[ref27] Lee J. Y. (2014). New and old roles of plasmodesmata in immunity and parallels to tunneling nanotubes. Plant Sci. 221–222, 13–20. 10.1016/j.plantsci.2014.01.006, PMID: 24656331 PMC4147083

[ref28] Lee J. Y. Wang X. Cui W. Sager R. Modla S. Czymmek K.. (2011). A plasmodesmata-localized protein mediates crosstalk between cell-to-cell communication and innate immunity in *Arabidopsis*. Plant Cell 23, 3353–3373. 10.1105/tpc.111.087742, PMID: 21934146 PMC3203451

[ref48] Letham D. S. Miller C. O. (1965). Identity of kinetin-like factors from Zea mays. Plant Cell Physiol. 6, 355–359. 10.1093/oxfordjournals.pcp.a079106

[ref29] Lim G. H. Shine M. B. De Lorenzo L. Yu K. Cui W. Navarre D.. (2016). Plasmodesmata localizing proteins regulate transport and signaling during systemic acquired immunity in plants. Cell Host Microbe 19, 541–549. 10.1016/j.chom.2016.03.006, PMID: 27078071

[ref30] Lucas W. J. Bouché-Pillon S. Jackson D. P. Nguyen L. Baker L. Ding B.. (1995). Selective trafficking of KNOTTED1 homeodomain protein and its mRNA through plasmodesmata. Science 270, 1980–1983. 10.1126/science.270.5244.1980, PMID: 8533088

[ref31] Mähönen A. P. Bishopp A. Higuchi M. Nieminen K. M. Kinoshita K. Törmäkangas K.. (2006). Cytokinin signaling and its inhibitor AHP6 regulate cell fate during vascular development. Science 311, 94–98. 10.1126/science.1118875, PMID: 16400151

[ref32] Müller B. Sheen J. (2007). Arabidopsis cytokinin signaling pathway. Sci. STKE 2007:cm5. 10.1126/stke.4072007cm5, PMID: 17925576

[ref33] Ormenese S. Bernier G. Périlleux C. (2006). Cytokinin application to the shoot apical meristem of *Sinapis alba* enhances secondary plasmodesmata formation. Planta 224, 1481–1484. 10.1007/s00425-006-0317-x, PMID: 16775701

[ref34] Peleg Z. Reguera M. Tumimbang E. Walia H. Blumwald E. (2011). Cytokinin-mediated source/sink modifications improve drought tolerance and increase grain yield in rice under water-stress. Plant Biotechnol. J. 9, 747–758. 10.1111/j.1467-7652.2010.00584.x, PMID: 21284800

[ref35] Rinne P. L. H. Van der Schoot C. (1998). Symplasmic fields in the tunica of the shoot apical meristem coordinate morphogenetic events. Development 125, 1477–1485. 10.1242/dev.125.8.1477, PMID: 9502728

[ref36] Roberts I. M. Boevink P. Roberts A. G. Sauer N. Reichel C. Oparka K. J. (2001). Dynamic changes in the frequency and architecture of plasmodesmata during the sink-source transition in tobacco leaves. Protoplasma 218, 31–44. 10.1007/BF01288358, PMID: 11732318

[ref37] Roberts A. G. Santa Cruz S. Roberts I. M. Prior D. A. M. Turgeon R. Oparka K. J. (1997). Phloem unloading in sink leaves of nicotiana benthamiana: comparison of a fluorescent solute with a fluorescent virus. Plant Cell 9, 1381–1396. 10.2307/3870389, PMID: 12237387 PMC157005

[ref38] Singh R. K. Miskolczi P. Maurya J. P. Bhalerao R. P. (2019). A tree ortholog of SHORT VEGETATIVE PHASE floral repressor mediates photoperiodic control of bud dormancy. Curr. Biol. 29, 128–133.e2. 10.1016/j.cub.2018.11.006, PMID: 30554900

[ref39] Stonebloom S. Burch-Smith T. Kim I. Meinke D. Mindrinos M. Zambryski P. (2009). Loss of the plant DEAD-box protein ISE1 leads to defective mitochondria and increased cell-to-cell transport via plasmodesmata. Proc. Natl. Acad. Sci. U. S. A. 106, 17229–17234. 10.1073/pnas.0909229106, PMID: 19805190 PMC2761335

[ref40] Turgeon R. (2010). The role of phloem loading reconsidered. Plant Physiol. 152, 1817–1823. 10.1104/pp.110.153023, PMID: 20200065 PMC2850027

[ref41] Tylewicz S. Petterle A. Marttila S. Miskolczi P. Azeez A. Singh R. K.. (2018). Photoperiodic control of seasonal growth is mediated by ABA acting on cell-cell communication. Science 360, 212–215. 10.1126/science.aan8576, PMID: 29519919

[ref42] Wang X. Sager R. Cui W. Zhang C. Lu H. Lee J. Y. (2013). Salicylic acid regulates plasmodesmata closure during innate immune responses in *Arabidopsis*. Plant Cell 25, 2315–2329. 10.1105/tpc.113.110676, PMID: 23749844 PMC3723628

[ref43] Werner T. Holst K. Pörs Y. Guivarc’h A. Mustroph A. Chriqui D.. (2008). Cytokinin deficiency causes distinct changes of sink and source parameters in tobacco shoots and roots. J. Exp. Bot. 59, 2659–2672. 10.1093/jxb/ern134, PMID: 18515826 PMC2486470

[ref44] Wybouw B. De Rybel B. (2019). Cytokinin – a developing story. Trends Plant Sci. 24, 177–185. 10.1016/j.tplants.2018.10.012, PMID: 30446307

[ref45] Xia Y. Li K. Li J. Wang T. Gu L. Xun L. (2019). T5 exonuclease-dependent assembly offers a low-cost method for efficient cloning and site-directed mutagenesis. Nucleic Acids Res. 47:e15. 10.1093/nar/gky1169, PMID: 30462336 PMC6379645

[ref46] Xu M. Cho E. Burch-Smith T. M. Zambryski P. C. (2012). Plasmodesmata formation and cell-to-cell transport are reduced in decreased size exclusion limit 1 during embryogenesis in *Arabidopsis*. Proc. Natl. Acad. Sci. U. S. A. 109, 5098–5103. 10.1073/pnas.1202919109, PMID: 22411811 PMC3324027

[ref47] Yadav R. K. Perales M. Gruel J. Girke T. Jönsson H. Venugopala Reddy G. (2011). WUSCHEL protein movement mediates stem cell homeostasis in the *Arabidopsis* shoot apex. Genes Dev. 25, 2025–2030. 10.1101/gad.17258511, PMID: 21979915 PMC3197201

